# The Role of Subjective Well-Being in the Relationship Between Illness Invalidation, Acceptance, and Functioning in Fibromyalgia

**DOI:** 10.3390/bs16020259

**Published:** 2026-02-10

**Authors:** Carlotta Sansone, Michael Tenti, Danilo Carrozzino, Paola Gremigni, Giulia Casu

**Affiliations:** 1 Department of Psychology, University of Bologna, 40127 Bologna, Italy; carlotta.sansone@studio.unibo.it (C.S.); danilo.carrozzino@unibo.it (D.C.); paola.gremigni2@unibo.it (P.G.); 2 ISAL Foundation, Institute for Research on Pain, 47921 Rimini, Italy; michael.tenti@fondazioneisal.it

**Keywords:** fibromyalgia, illness invalidation, illness acceptance, functioning, positive mental health, subjective well-being, mediation

## Abstract

Fibromyalgia (FM) is a chronic, widespread pain disorder that severely impairs daily functioning and well-being. Beyond physical symptoms, social and cognitive factors such as illness invalidation and difficulties in acceptance may further hinder adaptation. This study examined whether positive mental health and subjective well-being mediate the relationship between these factors and functioning in women with FM. A total of 148 women aged 20–65 completed self-report measures of the study variables. Preliminary correlation analyses showed that positive mental health was unrelated to invalidation and was therefore excluded from the mediation model. Mediation analyses revealed that higher illness invalidation and greater difficulties in illness acceptance were associated with lower subjective well-being, which in turn related to poorer functioning. These findings highlight the central role of subjective well-being in linking psychosocial factors to functioning in women with FM. Illness invalidation, acceptance difficulties, and subjective well-being itself emerged as key therapeutic targets, underscoring the importance of integrated, acceptance-based, and patient-centered interventions that foster well-being and support adaptive functioning despite persistent symptoms.

## 1. Introduction

In the realm of chronic widespread pain disorders, fibromyalgia (FM) has gained significant clinical attention from the scientific community due to its profoundly distressing and not yet fully understood nature. According to the ACTTION-APS Pain Taxonomy (AAPT) diagnostic criteria, FM is primarily diagnosed based on three core criteria: multisite chronic pain, along with sleep problems or fatigue, persisting for at least three months. Additional symptoms, such as musculoskeletal stiffness, environmental sensitivity, and cognitive difficulties, can complicate the clinical picture, leading to severe impacts on daily functioning (Arnold et al., 2019).

The negative effects of FM symptoms on patients’ lives are crucial areas of investigation to fully understand and manage this condition. Juuso et al. (2011) describe patients’ daily experiences with pain as unbearable and torturous, noting that the persistent cognitive and physical difficulties they must endure significantly impair their overall functioning. Many patients report having to reduce their working hours or change or even quit their jobs or education due to the debilitating effects of pain on their bodies and cognitive abilities (Arnold et al., 2008). Pain and fatigue are the symptoms that most significantly hinder work performance, and the severity of FM has been linked to decreased job productivity (Mukhida et al., 2020; Salaffi et al., 2022; Tenti et al., 2024b). Furthermore, pain hampers patients’ ability to care for themselves, their homes, and their families, leaving them with little energy for basic household chores (e.g., grocery shopping, house cleaning) and self-care activities (e.g., bathing, showering) (Arnold et al., 2008). Beyond daily responsibilities, pain also restricts their ability to travel, exercise, drive, and participate in sports, hobbies, and social gatherings (Arnold et al., 2008). Data on patient functioning contributes to research efforts aimed at understanding the impairments caused by chronic pain related to FM. Functioning is a critical clinical domain valued by both patients and clinicians, serving as a core outcome measure in both clinical research (e.g., drug or psychotherapy) trials and practice (Choy, 2010; Wolfe et al., 2011). Its assessment is essential for providing holistic care, improving treatment outcomes, and enhancing the quality of life for those affected by FM.

When investigating functioning in patients with FM, it is crucial to identify and address the factors that influence the impact of pain on daily activities.

### 1.1. Invalidation and Its Effects on Functioning and Well-Being

Illness invalidation is an emerging construct in health psychology, encompassing both active negative social responses (e.g., denying and discounting) and a lack of positive social responses (e.g., supporting and acknowledging) towards patients and their conditions (Kool et al., 2009). This experience, which was defined for the first time by Kool et al. (2009), entails the perception of cognitive, affective, and behavioral responses of others that are perceived as denying, lecturing, overprotecting, not supporting and not acknowledging with respect to the clinical condition of the patient. Illness invalidation is particularly significant in FM, given that symptoms of this clinical condition (e.g., pain, fatigue, and stiffness) are inherently invisible (Ghavidel-Parsa et al., 2021). The most severe consequences of illness invalidation are indeed found among FM patients, as the absence of clear physical evidence of their suffering can create a significant discrepancy between the patients’ perception of their condition and that of their social environment (Santiago et al., 2017). Invalidation can come from various sources within the social environment, including spouses, the workplace, and social services. According to Santiago et al. (2017), individuals with rheumatic diseases often experience more invalidation from family members than from health professionals. However, the invalidation perceived from health professionals should not be overlooked, as it can negatively impact clinical encounters and treatment outcomes (Santiago et al., 2017). Key dimensions of invalidation, such as discounting and lack of understanding, can lead to feelings of loneliness (Kool & Geenen, 2013) and poorer physical and mental health (Kool et al., 2013), as well as increased FM symptom severity and negative health outcomes (Ghavidel-Parsa et al., 2015). The lack of recognition or understanding from others can lead to feelings of isolation, distress, and a sense of being unsupported. When patients feel their condition is not validated, this can exacerbate symptoms and impair daily functioning (Galvez-Sánchez et al., 2019). In fact, Järemo et al. (2022) revealed that patients who do not experience any form of invalidation report better quality of life, less pain, and higher satisfaction with care. On the other hand, patients who do experience invalidation employ a considerable amount of energy to make others aware and understanding of their condition (Järemo et al., 2022). Therefore, invalidation’s detrimental effects are evident in patients’ functioning, creating significant hurdles in managing everyday life and coping with the ongoing lack of understanding from others (Ghavidel-Parsa et al., 2021).

The psychological flexibility model, which underlies Acceptance and Commitment Therapy (ACT), provides a valuable conceptual framework for understanding the negative impact of illness invalidation on functioning in individuals with FM. Psychological flexibility is defined as the ability to persist in or modify behavior in pursuit of personally valued goals, even in the presence of difficult internal experiences (Hayes et al., 2006). When people feel their pain is invalidated, they might react by either striving to prove their condition’s legitimacy or withdrawing from social interactions (Nicola et al., 2021; Paxman, 2021). These processes can contribute to experiential avoidance, wherein individuals attempt to resist, suppress, or control distressing emotions and pain-related thoughts (McCracken & Morley, 2014), as well as the social consequences of invalidation. From an ACT perspective, rigid responses to invalidation—such as persistent efforts to seek external validation or suppress emotional distress—may consume psychological resources that could otherwise be directed toward meaningful life activities, ultimately impairing functioning. In contrast, fostering psychological flexibility allows patients to acknowledge the presence of pain and social invalidation without letting these experiences dictate their actions. Empirical evidence supports this link. Cameron et al. (2018) found a negative association between invalidation and pain acceptance in individuals with rheumatic diseases, suggesting that those who experience greater invalidation may struggle more with accepting their pain. This finding underscores the central role of pain acceptance in mitigating the adverse effects of invalidation, thereby promoting functioning and well-being in FM patients.

### 1.2. Acceptance and Its Effects on Functioning and Well-Being

Acceptance involves acknowledging and coming to terms with the chronic nature of FM and its symptoms without attempting to avoid or deny them (Lachapelle et al., 2008). Higher levels of pain acceptance have been associated with higher functioning and better psychological adjustment to pain (McCracken & Eccleston, 2005; Trainor et al., 2019; Tangen et al., 2020). Acceptance is associated with better physical functioning, based on the evidence that patients who accept their pain are more likely to participate in physical activities, which can help manage symptoms and improve overall functioning and health. Of note, lower pain acceptance has been associated with poorer physical functioning assessed with both self-reported and performance-based measures (Varallo et al., 2021). As illustrated by Tangen et al. (2020), by developing pain acceptance, patients can learn to positively deal with their condition, identifying valued directions and orienting towards the construction of a life between moments of dysfunction (Rodero et al., 2011). In the long run, this process is linked to better day-to-day functioning and improvements in physical, emotional, and social condition (Rodero et al., 2011). Acceptance helps patients focus on positive aspects of their lives, which can enhance emotional well-being and lead to a more positive outlook on life (Viane et al., 2003). Accepting pain also reduces psychological distress, leading to lower levels of anxiety and depression as patients stop fighting against their pain (Luciano et al., 2014; Wicksell et al., 2012). Finally, by accepting their condition, patients can engage more fully in daily activities and social interactions, providing a sense of purpose and fulfillment (Hanel et al., 2024).

### 1.3. Effects of Fibromyalgia on Mental Health and Subjective Well-Being

Research has demonstrated that, besides functioning, all three main dimensions of mental health—emotional, social, and psychological—are adversely affected by FM. Keyes (2002) developed a comprehensive definition of positive mental health by integrating the psychological and social aspects of eudaimonic well-being with the cognitive and affective components of hedonic well-being. The eudaimonic perspective focuses on optimal functioning in life, considering two key aspects: psychological well-being (Ryff, 1989) and social well-being (Keyes, 2002). Psychological well-being primarily involves self-actualization, including having a sense of purpose in life, achieving personal goals, fostering growth, and effectively managing life. Social well-being, on the other hand, relates to how individuals perceive their roles and functioning within society, including evaluating their sense of social contributions, belonging, acceptance, and support. In contrast, the hedonic approach focuses on the subjective experience of pleasure, emphasizing positive emotional states, happiness, life satisfaction, and the avoidance of pain (Deci & Ryan, 2008; Ryff et al., 2021). Within the hedonic perspective, the World Health Organization defines subjective well-being as a global evaluation of one’s own life in terms of positive emotional experiences and optimal functioning (Topp et al., 2015).

In a study on women with chronic musculoskeletal pain, Huber et al. (2008) found that both hedonic and eudaimonic components of well-being decreased with higher disability, independently of other aspects like age or pain intensity. FM has been found to have a more pronounced impact on different dimensions of well-being compared to other pain conditions (Boonen et al., 2005; Kaplan et al., 2000; Schleicher et al., 2005). Consistent with these findings, research indicates that FM significantly affects both eudaimonic and hedonic well-being, further underscoring its widespread impact on mental health (Galvez-Sánchez et al., 2019).

### 1.4. Effects of Eudaimonic and Hedonic Well-Being on Functioning

A decline in aspects of well-being can, in turn, negatively affect overall functioning (Galvez-Sánchez et al., 2019). Extensive research has explored various predictors of impaired functioning in patients with FM. Among these, eudaimonic and hedonic well-being could play a significant role, though this area remains relatively unexplored in the literature. Schleicher et al. (2005) found that greater eudaimonic well-being was associated with better overall functioning, though not with reduced pain. Pennato et al. (2011) identified two dimensions of eudaimonic well-being—purpose in life and positive relationships—as predictors of functional impairment in FM, beyond potential confounding variables such as age, marital status, education, anxiety, and depression. Schleicher et al. (2005) further noted that individuals with high scores in well-being dimensions of self-acceptance, environmental mastery, purpose in life, and positive relationships tend to have a positive self-image, effective coping skills, a sense of direction, and fulfilling social connections. These qualities may help them function effectively despite FM-related challenges.

Despite extensive research on the predictors of impaired functioning in FM patients, a gap remains in the literature. Specifically, there is a lack of studies investigating how cognitive and behavioral factors interact with positive mental health and subjective well-being within the clinical profiles of FM patients, affecting their daily functioning.

**Figure 1:**
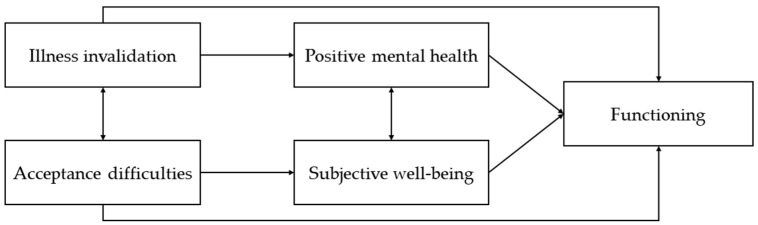
The Proposed Mediation Model.

The present study aims to address this research gap by exploring whether positive mental health—as a combination of hedonic and eudemonic aspects (Keyes, 2002), and subjective well-being—within the hedonic approach (Topp et al., 2015), serve as mediators in the relationship between cognitive factors (illness invalidation and acceptance difficulties) and overall functioning, as illustrated in Figure 1. By investigating these factors, we aim to uncover how they influence the functioning of individuals with FM. Understanding the associations among predictors, mediators, and outcomes is crucial for developing comprehensive treatment approaches that address both the physical and psychological dimensions of FM. This approach goes beyond alleviating pain and suffering, aiming to promote positive adjustment to the condition. By fostering psychological resilience, patients may be better equipped to leverage their resources and pursue their aspirations despite chronic pain.

In testing this mediation model, we also considered potential confounding sociodemographic and clinical variables that could influence the main variables based on the literature, such as age (e.g., Jiao et al., 2014), educational level (e.g., Atzeni et al., 2022; Fentazi et al., 2025), employment (e.g., Muller et al., 2017), marital status (e.g., Atzeni et al., 2022), having children (e.g., Lo Monaco et al., 2024), duration of symptoms (e.g., Gendelman et al., 2018; Häuser et al., 2011), and time to diagnosis (e.g., Gendelman et al., 2018; Häuser et al., 2011; Salaffi et al., 2024).

## 2. Materials and Methods

### 2.1. Study Design and Procedure

This study adopts a cross-sectional research design, with a single administration of an online survey to women recruited via the Internet from several forums dedicated to FM. To facilitate distribution, forum moderators were asked to share a brief description of the research purposes along with a survey link. Upon access, participants received a detailed study overview and provided informed consent to participate anonymously. Eligibility criteria were being over 18 years old and reporting a diagnosis of FM. The study was approved by the Bioethics Committee of the University of Bologna (protocol number 0026440, 2 February 2023).

### 2.2. Measures

#### 2.2.1. Sociodemographic and Illness-Related Characteristics

The first section of the survey collected information on socio-demographic and FM-related characteristics, which included age, education level, employment status, marital status, and motherhood status. FM-related characteristics included duration of symptoms and time to diagnosis.

Validated self-report measures were employed to assess the variables included in the proposed conceptual model, including illness invalidation, illness acceptance difficulties, positive mental health, subjective well-being, and FM-related functioning. Measures not available in Italian were translated and back-translated by two independent bilingual professionals, following international guidelines for test translation and cross-cultural adaptation (Hambleton et al., 2005).

#### 2.2.2. The Illness Invalidation Inventory (3*I)

The 3*I (Kool et al., 2010) measures perceived invalidation of pain from five important sources: partner, family, medical professionals, work environment, and social services. The same 8 items are presented for each source, for a total of 40 items. Responses are rated on a 5-point scale (1 = never to 5 = very often), indicating how frequently individuals perceive specific reactions from each source. Factor analyses identified two factors for each source: Discounting, which reflects negative social responses such as disbelief or dismissal (5 items; e.g., “…finds it odd that I can do much more on some days than on other days”), and Lack of understanding, representing the absence of supportive or validating responses (3 reverse items; e.g., “…takes me seriously”). Higher scores in each factor and in the total 3*I indicate greater illness invalidation. In the present study, a total mean invalidation score was computed by averaging scores across the five sources, providing an overall indicator of perceived illness invalidation. Cronbach’s α coefficients indicated good internal consistency across sources, ranging from 0.83 (work environment) to 0.89 (social services).

#### 2.2.3. The Acceptance of Illness Scale (AIS)

The AIS (Felton & Revenson, 1984) was originally developed to measure acceptance of illness, which reflects the adaptation to new life conditions and changes in quality of life due to the onset of an illness, in a sample of middle-aged and older adults with one of four chronic illnesses (Czerw et al., 2022; Felton & Revenson, 1984). The measure is composed of 8 statements (e.g., “I have problems with adapting to limitations imposed by my illness”), with respondents rating their agreement on a 5-point scale from 1 (strongly disagree) to 5 (totally agree). Higher scores correspond to lower levels of illness acceptance and adaptation, along with a greater sense of psychological discomfort. Rankin and Holttum (2003) adapted the scale for pain acceptance by replacing “illness” with “pain” in each item, and this adaptation showed acceptable internal consistency, with a Cronbach’s α of 0.78. Cronbach’s α in this study was 0.87.

#### 2.2.4. The Mental Health Continuum–Short Form (MHC–SF)

The MHC–SF (Keyes, 2002; Keyes et al., 2008) is a short version of the 40-item Mental Health Continuum-Long Form (MHC-LF; Keyes, 2002), designed to measure emotional, social, and psychological well-being (Petrillo et al., 2015). Its 14 items are arranged into three subscales: Emotional well-being (3 items; e.g., “During the past month, how often did you feel interested in life”), Social well-being (5 items; e.g., “During the past month, how often did you feel that you had something important to contribute to society”), and Psychological well-being (6 items; e.g., “During the past month, how often did you feel that you liked most parts of your personality”), with responses rated on a 6-point scale from 0 (none of the time) to 5 (all of the time). The MHC-SF integrates hedonic well-being (positive emotional states) with eudaimonic well-being (optimal social and psychological functioning) to provide a comprehensive view of positive mental health (Keyes, 2002). Petrillo et al. (2015) validated the Italian version, confirming the three-factor structure and showing good psychometric properties, including adequate reliability for the total scale (α = 0.86) and the subscales (α values between 0.70 and 0.81). In this study, Cronbach’s α was 0.94 for the total scale and between 0.84 and 0.91 for the subscales. Since there was evidence of a second-order single factor (Petrillo et al., 2015) and intercorrelations between factors were strong in this study (Pearson’s *r*s between 0.66 and 0.82, *p* < 0.001), we used the total scale as a single variable named Positive Mental Health (PMH).

#### 2.2.5. The 5-Item World Health Organization Well-Being Index (WHO-5)

The WHO-5 (World Health Organization, 1998; Topp et al., 2015) is a short 5-item scale derived from the 28-item version employed in a WHO multicenter study across eight European countries (Johansen, 1989). The WHO-5 assesses positive subjective well-being within the hedonic framework (Blom et al., 2012). Responses are rated on a 6-point scale, from 0 (none of the time) to 5 (all the time), where the respondent indicates how often each item (e.g., “Over the past 2 weeks I have felt cheerful and in good spirits”) applied to them over the past two weeks. The one-factor model was validated by Krieger et al. (2014), and Birket-Smith et al. (2009) proved its predictive validity. Bech et al. (2003) reported adequate reliability (α = 0.84), and Topp et al. (2015) reported satisfactory sensitivity (0.86) and specificity (0.81) for depression screening. The WHO Regional Office in Europe translated the scale into over 30 languages, including Italian (Topp et al., 2015). In this study, Cronbach’s α was 0.81. We named the variable measured by the WHO-5 Subjective Well-being (SWB).

#### 2.2.6. The Revised Fibromyalgia Impact Questionnaire (FIQR)

The FIQR (Bennett et al., 2009) was developed to overcome the limitations of the Fibromyalgia Impact Questionnaire (FIQ; Burckhardt et al., 1991), the most used tool for evaluating FM patients. The FIQR is a 21-item measure covering the three domains of Function (9 items, e.g., “Brush or comb hair”), Impact (2 items, e.g., “Cannot achieve goals”), and Symptoms (10 items, e.g., “Pain rating”). Function refers to daily life activities; Impact refers to the overall impact of FM on functioning in terms of not being able to achieve goals and feeling overwhelmed; and Symptoms contain questions related to rating of pain, energy, sleep quality, etc. All items are based on an 11-point Likert scale answered from 0 (no difficulties) to 10 (extreme difficulties) for Function, from 0 (never) to 10 (always) for Impact and from 0 (symptom’s absence) to 10 (extreme symptom’s presence) for Symptoms. Higher scores are indicative of greater dysfunction or symptom severity. The Italian version validated by Salaffi et al. (2013) was used in this study, demonstrating structural and convergent validity and reliability. Cronbach’s α in the current study indicated adequate internal consistency for the Function (α = 0.91), Impact (α = 0.88), and Symptoms (α = 0.81) subscales.

### 2.3. Data Analysis

Descriptive statistics were computed to summarize participants’ characteristics and psychological variable scores.

Based on the proposed conceptual model (Figure 1), illness invalidation and illness acceptance difficulties were specified as predictor variables, positive mental health and subjective well-being as potential mediators, and FM-related functioning (FIQR Function, Impact, and Symptoms) as outcome variables.

To ensure the absence of multicollinearity, Pearson’s correlation coefficients were computed among predictors, among mediators, and between predictors and mediators. Correlation coefficients below *r* = 0.80 were considered acceptable (Young, 2017). Preliminary Pearson’s correlation analyses were then conducted to verify that the proposed mediators (i.e., Positive Mental Health and Subjective Well-Being) were significantly associated with both the predictor and outcome variables, as well as to identify potential covariates to be included in the mediation model. Sociodemographic variables (age, education, marital status, children, and employment status) and FM-related variables (years with symptoms and time to diagnosis) were included as covariates if they significantly correlated with the mediator and/or outcome variables at *r* ≥ |0.30| (Frigon & Laurencelle, 1993).

The mediation model was tested using mediation analysis with the maximum likelihood estimator. Indirect effects were estimated and tested using bootstrapping with 1000 resamples (Preacher & Hayes, 2008). Bias-corrected 95% bootstrapped confidence intervals that did not include zero were considered statistically significant.

Significance level was set at *p* ≤ 0.05. All analyses were conducted with JASP version 0.18.3.

## 3. Results

### 3.1. Participants

The sample included 148 women with a diagnosis of FM. Regarding sociodemographic characteristics (Table 1), the mean age was about 49 years. Most participants had a high school or university degree, were married or cohabitating, and had children. Approximately half of them were employed, students, or working students. The duration of FM symptoms, defined as the number of years since initial complaints, ranged from less than one year to 50 years, whereas time to FM diagnosis, defined as the number of years between symptom onset and receiving a diagnosis, ranged from less than one year to 42 years. These characteristics were generally not largely different from those of other studies (e.g., Häuser et al., 2011; Martini et al., 2019; Tenti et al., 2024a).

### 3.2. Preliminary Analyses

As shown in Table 2, correlations among predictors and mediators were all below *r* = 0.80, thus ruling out potential multicollinearity issues.

Positive Mental Health was significantly and negatively correlated with all three dimensions of functioning, as well as with acceptance difficulties, but was unrelated to illness invalidation. Conversely, Subjective Well-Being was significantly and negatively correlated with the three dimensions of functioning and with both predictors. Therefore, only Subjective Well-Being was included in mediation analyses.

Regarding potential confounding variables (see Appendix A), only employment status showed significant, moderate correlations with the outcome variables (Function: *r* = −0.28, *p* < 0.001; Impact: *r* = −0.33, *p* < 0.001; Symptoms: *r* = −0.25, *p* = 0.002) and was therefore controlled for in the mediation model.

### 3.3. Mediation Analyses

**Figure 2:**
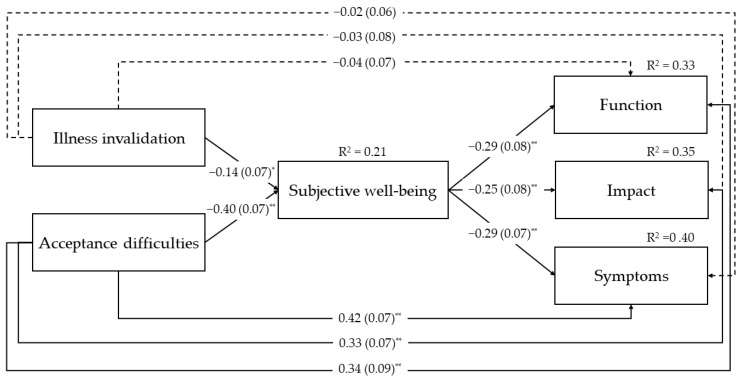
Tested Mediation Model. Standardized estimates (standard errors) are reported. Dashed lines indicate nonsignificant effects. Correlation between the predictors, covariances between the outcome variables, and the effects of the covariate (employment) were estimated but are not shown to improve readability. * *p* ≤ 0.05. ** *p* ≤ 0.001.

As shown in Figure 2, higher illness invalidation and greater acceptance difficulties were both significantly associated with lower subjective well-being. Lower subjective well-being, in turn, was significantly associated with poorer performance in daily activities (Function), greater perceived impact of FM on goal achievement and emotional overwhelm (Impact), and more severe symptom-related problems (Symptoms).

All indirect effects were significant (Table 3), indicating that higher levels of illness invalidation and acceptance difficulties were indirectly related to poorer functioning across all three dimensions through lower subjective well-being.

The direct effects of illness invalidation were nonsignificant for all three dimensions of functioning, indicating full mediation, whereas the direct effects of acceptance difficulties were significant, indicating partial mediation (Figure 2).

Regarding the effects of the covariate (employment status), being employed, studying, or being a working student was associated with higher perceived invalidation (*b* = 0.18, *SE* = 0.08, *z* = 2.15, *p* = 0.03), lower acceptance difficulties (*b* = −0.27, *SE* = 0.08, *z* = −3.32, *p* < 0.001), better performance in daily activities (*b* = −0.15, *SE* = 0.07, *z* = 2.00, *p* = 0.046), and a lower perceived impact of FM on goal achievement and emotional overwhelm.

The model explained 21% of the variance in subjective well-being, 33% in Function, 35% in Impact, and 40% in Symptoms.

## 4. Discussion

Findings from the present study indicated that illness invalidation and acceptance difficulties showed significant direct associations with overall functioning of patients with FM, with subjective well-being serving as a full mediator in the former relationship and a partial mediator in the latter.

In the case of illness invalidation, the absence of a direct association with functioning, combined with the significant indirect association through subjective well-being, suggests that the association between invalidation and participants’ daily lives is statistically accounted for by their well-being. Experiences of being disbelieved, dismissed, or misunderstood may not directly impair one’s ability to perform daily activities, but they can erode emotional balance, self-worth, and overall life satisfaction (e.g., Kool & Geenen, 2013; Ghavidel-Parsa et al., 2021). This reduction in subjective well-being, in turn, may lead to disengagement from meaningful activities and greater perceived functional limitations (Galvez-Sánchez et al., 2019). From a theoretical perspective, these results underscore the role of subjective well-being as a psychological process between the social experience of invalidation and poorer functioning. This interpretation is consistent with previous findings indicating that invalidation is closely linked to emotional distress and diminished well-being among individuals with chronic pain (Kool & Geenen, 2013; Järemo et al., 2022; Galvez-Sánchez et al., 2019).

The direct and indirect associations between acceptance difficulties and poorer functioning are consistent with theoretical frameworks emphasizing acceptance as a key psychological resource in chronic pain management (e.g., ACT; McCracken & Vowles, 2014). Acceptance may facilitate better functioning by reducing resistance to pain and perceived limitations, thereby allowing individuals to engage more fully in valued activities and to remain active in meaningful life domains despite symptoms (McCracken & Eccleston, 2003). The direct association of acceptance difficulties with overall functioning observed in the present study is in line with previous findings. Several authors (e.g., McCracken & Eccleston, 2005; Tangen et al., 2020; Trainor et al., 2019) have reported that higher levels of pain acceptance are associated with greater functioning and better psychological adjustment to pain, while Rodero et al. (2011) found that low acceptance directly contributes to increased pain, distress, and disability. Our mediation analysis extends this evidence by identifying an indirect pathway between acceptance difficulties and functioning through subjective well-being. The partial mediation observed suggests that acceptance is associated with better functioning not only directly, but also through more positive emotional and cognitive evaluations of one’s life. This interpretation is in line with positive psychology perspectives, which propose that well-being can buffer the negative impact of chronic stressors on daily functioning (Hu & Gruber, 2008; Diener & Chan, 2011; Diener et al., 2017).

Interestingly, positive mental health was not significantly associated with illness invalidation and was therefore not included in the mediation model. This lack of association may be explained by conceptual differences between positive mental health and subjective well-being. While subjective well-being captures more immediate, affective, and situational evaluations of one’s life—often fluctuating in response to daily experiences and interpersonal stressors—positive mental health represents a broader and more stable construct encompassing psychological, emotional, and social well-being (Keyes, 2002). As such, positive mental health may be less affected by short-term interpersonal dynamics that characterize experiences of illness invalidation. Accordingly, patients with FM may experience illness invalidation while still maintaining relatively high levels of positive mental health. This is consistent with the concept of positive mental health as a distinct indicator of optimal psychosocial functioning that is related to, but different from, mental illness (Keyes, 2002). According to this model, individuals may experience psychological suffering while simultaneously maintaining positive mental health (Keyes, 2002). This idea is conceptually rooted in the eudaimonic tradition, which considers positive mental health as a dynamic and self-actualizing process that supports meaning-making, personal growth, and values-oriented goal pursuit despite ongoing physical and mental suffering (Deci & Ryan, 2008; Giuntoli et al., 2021; Ryff et al., 2021). In contrast to the hedonic perspective, which primarily focuses on subjective well-being as a state related to the presence of pleasant emotions, the eudaimonic approach emphasizes more enduring psychological resources that are less directly shaped by momentary interpersonal experiences.

In this sense, positive mental health reflects deeper psychological resources, such as self-acceptance, purpose in life, or social integration, that are more closely linked to internal processes of adaptation to illness and broader indicators of long-term functioning than to the immediate emotional impact of invalidating interpersonal experiences. By contrast, patients’ perceptions of being disbelieved or misunderstood are likely to influence their emotional state and daily satisfaction more directly, which may explain why subjective well-being, but not positive mental health, emerged as a significant mediator between illness invalidation and functioning. While eudaimonic dimensions of well-being have been associated with long-term adjustment and adaptive coping (Giuntoli et al., 2021), their effects may unfold more gradually and may not fully capture the proximal, emotion-driven responses triggered by social invalidation. Future longitudinal research could therefore clarify whether positive mental health is associated with functioning through slower, resilience-based mechanisms rather than through immediate affective responses.

In this study, being employed, studying, or combining both roles was associated with higher perceived illness invalidation, lower difficulties in illness acceptance, and better functioning and lower perceived impact of FM symptoms. The association between employment and lower FIQR scores aligns with previous research showing that individuals who remain active through work or study tend to report better physical and psychosocial functioning (Palstam & Mannerkorpi, 2017; Tangen et al., 2020). This relationship may be bidirectional: individuals with fewer functional limitations may be more capable of maintaining work or study roles, while engagement in these activities could, in turn, enhance perceived competence and promote a sense of normalcy despite ongoing symptoms.

Interestingly, employed or studying participants also reported greater illness invalidation. This might reflect the higher social exposure and greater expectations encountered in professional or academic environments, where invisible symptoms are often questioned or underestimated (Santiago et al., 2017). However, despite perceiving more invalidation, these individuals showed fewer difficulties in accepting their illness, suggesting that continued engagement in valued roles may facilitate psychological flexibility and acceptance processes. Maintaining work or study routines may thus represent both a source of social challenge and a means of fostering adaptive coping. Further research is warranted to explore how occupational and educational engagement simultaneously shape perceived invalidation, acceptance, and functional outcomes in FM.

This study is not without limitations. The first limitation concerns its cross-sectional design, which inherently limits the temporal scope of the findings. Moreover, all data were obtained through self-report instruments, which rely on participants’ subjective evaluations and retrospective recall. Such measures are inherently vulnerable to memory biases, mood-dependent distortions, and fluctuations in perception over time in response to both internal and external factors. Consequently, the results capture a momentary snapshot of participants’ experiences rather than the dynamic and evolving nature of their illness adaptation. Future studies would benefit from adopting longitudinal designs, enabling researchers to track changes in perceptions, emotions, and functioning over time. These approaches could provide a more nuanced understanding of the temporal evolution of patients’ experiences, as well as clearer insights into causal and contextual mechanisms underlying the observed associations. In addition, the exclusive reliance on self-report measures collected at a single time point raises the possibility of common method bias, which may have inflated the observed associations due to shared method variance, response styles, or transient affective states. Future research would benefit from incorporating multiple sources of data, such as objective or performance-based measures of functioning, clinician-rated outcomes, or reports from significant others. Moreover, multi-wave or multi-method designs could help disentangle substantive relationships among variables from method-related artifacts and provide a more robust assessment of the proposed associations (Podsakoff et al., 2024).

An additional limitation concerns the composition of the sample, which consisted exclusively of women. The absence of male participants limits the generalizability of the findings to the broader population of individuals with FM. Existing literature suggests that some sex differences may be present in FM, for example, in pain characteristics, functional impairment, and the ways individuals think about and cope with pain (Favretti et al., 2023; Racine et al., 2015). However, findings in this area are mixed, and it remains unclear whether and to what extent such differences are robust and clinically meaningful. For this reason, future studies should aim to recruit larger and more sex-balanced samples in order to systematically examine potential sex-related differences and to improve the external validity of the findings.

A further limitation concerns the absence of key clinical covariates in the mediation model. In the present study, illness invalidation and acceptance difficulties were examined as predictors of overall functioning through subjective well-being. However, functioning in FM is also strongly influenced by clinical variables such as pain intensity, fatigue severity, and emotional comorbidities (e.g., anxiety and depression) (Montoro & Galvez-Sánchez, 2022; Steiner et al., 2017), which were not included in the model. The omission of these factors may have influenced the observed associations and limited the ability to fully isolate the indirect effects through subjective well-being. Future studies should therefore integrate illness invalidation, acceptance and subjective well-being within more comprehensive models that also account for relevant clinical and emotional covariates in order to better delineate their unique and incremental contributions to overall functioning.

Another potential limitation concerns the data collection procedure, which relied on an online survey. This approach offers many advantages, such as cost-effectiveness, efficiency, and broad geographical reach, but also presents certain drawbacks, such as limited sample representativeness due to the possible exclusion of individuals without Internet access, self-selection bias among participants particularly interested in the study topic, and lack of control over environmental conditions that may affect the quality and accuracy of responses. Moreover, while anonymity can encourage openness and honesty, it may also increase the likelihood of inattentive or careless responding. To mitigate these issues, future studies could incorporate attention checks to identify low-quality responses, include items assessing impression management or social desirability, and combine online quantitative surveys with qualitative methods (e.g., interviews).

A final limitation concerns the recruitment strategy, as the sample was drawn from online forums focused on a specific topic. Consequently, it may not be fully representative of the broader population of women with FM. Individuals who participate in these forums may be more actively engaged with their illness, experience a higher psychosocial burden, or be more attuned to interpersonal experiences such as illness invalidation. This recruitment strategy may therefore result in an overrepresentation of specific viewpoints and reduced variability within the sample, limiting the generalizability of the findings to women with FM who are less active in online communities or who are recruited in clinical or community-based settings. Future research should consider using multiple recruitment channels to increase sample diversity or applying stratified sampling methods to ensure adequate representation across key demographic and clinical characteristics.

## 5. Conclusions

Findings from the present study are consistent with recent literature emphasizing the crucial role of acceptance-based interventions in improving functioning among individuals with FM (Eastwood & Godfrey, 2024). Such approaches have also been endorsed by the NICE guidelines for the management of primary chronic pain, including FM, as evidence-based strategies to help patients cope with persistent symptoms (NICE Guideline NG193, 2021). Our results further suggest that the effectiveness of such interventions may be strengthened by incorporating components that specifically target subjective well-being, such as the enhancement of positive emotions, reduction in distress, and improvement of life satisfaction. Fostering these domains could promote greater resilience and adaptive coping, ultimately supporting patients in pursuing a meaningful and fulfilling life despite the persistence of symptoms.

Our findings also emphasize the association between overall illness invalidation and patients’ well-being and functioning. Clinicians should be aware of the detrimental effects that feeling disbelieved or misunderstood can have on patients’ emotional adjustment and engagement in treatment. Fostering a therapeutic and social environment that validates patients’ experiences may promote adjustment and active participation in care. Training healthcare professionals to recognize and counteract invalidating attitudes may further improve communication, strengthen trust, and enhance adherence to therapeutic recommendations.

Employment and study roles might act as protective factors for individuals with FM, underscoring the importance of interventions aimed at supporting job retention and academic participation. The literature offers several recommendations for reasonable workplace accommodations that can enable individuals with FM to maintain employment and productivity (Tenti et al., 2024b). Evidence also indicates that when the work environment is supportive and appropriately adapted to individual needs, people with FM can remain employed and experience job satisfaction despite ongoing symptoms (Henriksson et al., 2005).

Overall, this study contributes to the understanding of the complex pathways linking psychosocial factors to functioning in women with FM. It highlights the relevance of illness invalidation, acceptance difficulties, and subjective well-being as potential therapeutic targets and underscores the importance of developing integrated, patient-centered interventions that account for both individual and contextual dimensions of illness experience. At the same time, given the cross-sectional nature of the study, the proposed mediation model should be interpreted as a theoretically informed representation of the associations among key psychosocial variables rather than as evidence of definitive causal pathways. As such, our findings should be viewed as hypothesis-generating and warrant confirmation through future longitudinal research aimed at establishing temporal precedence and determining whether changes in these psychosocial factors lead to subsequent improvements in functioning.

## Figures and Tables

**Table 1:** Socio-demographic and clinical characteristics of the participants.

Characteristic	N	%
Age, M, SD, range	48.82	10.37, 20–65
Education		
Up to middle school	38	25.7
High school or degree/post-degree	110	74.3
Marital status		
Unmarried/separated/widowed	52	35.1
Married/cohabiting	96	64.9
Having children	105	70.9
Employment status		
Unemployed/housemaker/retired/other	66	44.6
Employed/student/working student	82	55.4
Duration (years with FM symptoms), M, SD, range	14.86	11.74, 0–50
Years to FM diagnosis, M, SD, range	8.86	9.85, 0–42

FM = fibromyalgia; M = mean; SD = standard deviation.

**Table 2:** Descriptive statistics and Pearson’s correlations among the study variables.

	1	2	3	4	5	6	7
1. Illness invalidation	-						
2. Acceptance difficulties	0.23 **	-					
3. PMH	−0.09	−0.34 ***	-				
4. SWB	−0.23 **	−0.44 ***	0.48 **	-			
5. FIQR-Function	0.08	0.50 ***	−0.21 **	−0.45 ***	-		
6. FIQR-Impact	0.13	0.51 ***	−0.17 *	−0.43 ***	0.68 ***	-	
7. FIQR-Symptoms	0.16 *	0.58 ***	−0.23 **	−0.49 ***	0.69 ***	0.69 ***	-
M	22.65	28.48	26.65	31.46	20.72	13.88	37.15
SD	5.09	7.16	15.16	16.82	5.74	4.83	6.59

PMH = Positive Mental Health; SWB = Subjective Well-Being; FIQR = Fibromyalgia Impact Questionnaire—Revised; M = mean; SD = standard deviation. * *p* ≤ 0.05. ** *p* ≤ 0.01. *** *p* ≤ 0.001.

**Table 3:** Indirect effects.

Indirect Effect	*b*	*SE*	95% CI
Illness invalidation → Subjective well-being → FIQR-Function	0.04	0.03	[0.003, 0.104]
Illness invalidation → Subjective well-being → FIQR-Impact	0.04	0.02	[0.004, 0.102]
Illness invalidation → Subjective well-being → FIQR-Symptoms	0.04	0.03	[0.003, 0.107]
Acceptance difficulties → Subjective well-being → FIQR-Function	0.12	0.04	[0.047, 0.201]
Acceptance difficulties → Subjective well-being → FIQR-Impact	0.10	0.04	[0.039, 0.178]
Acceptance difficulties → Subjective well-being → FIQR-Symptoms	0.11	0.03	[0.055, 0.178]

*SE* = standard error; CI = confidence interval; FIQR = Fibromyalgia Impact Questionnaire—Revised. Standardized estimates and bootstrapped CIs are reported.

## Data Availability

The data presented in this study are available on request from the corresponding author due to privacy restrictions.

## Appendix A

**Table A1:** Correlations between sociodemographic/clinical characteristics and the study variables.

	PMH	SWB	FIQR-Function	FIQR-Impact	FIQR-Symptoms
Age	−0.06	0.04	0.10	0.04	0.13
Education ^1^	−0.02	−0.10	−0.18 *	−0.22 **	−0.21 *
Employment status ^2^	0.05	0.12	−0.28 ***	−0.33 ***	−0.25 **
Marital status ^3^	0.02	0.11	0.02	−0.07	0.02
Having children ^4^	0.09	0.05	0.17 *	0.03	0.17 *
Years with symptoms	−0.01	0.15	0.10	0.06	0.11
Years since diagnosis	−0.01	0.05	0.12	0.10	0.14

^1^ high school/degree/post-degree coded 1. ^2^ employed/student/working student coded 1. ^3^ married/cohabiting coded 1. ^4^ yes coded 1. PMH = Positive Mental Health; SWB = Subjective Well-Being; FIQR = Fibromyalgia Impact Questionnaire—Revised; * *p* ≤ 0.05. ** *p* ≤ 0.01. *** *p* ≤ 0.001.
